# Restoration of hip geometry after total hip arthroplasty: retrospective comparison of two short stems and one straight stem

**DOI:** 10.1186/s12891-022-05923-4

**Published:** 2022-11-30

**Authors:** Werner Maurer-Ertl, Joerg Friesenbichler, Michael Pfann, Michael Maier, Patrick Reinbacher, Andreas Leithner, Maria A. Smolle

**Affiliations:** 1 Department of Orthopaedics and Trauma, Medical University of Graz, Auenbruggerplatz 5, 8036 Graz, Austria; 2 General Public Hospital Guessing, Grazer Straße 15, 7540 Guessing, Austria

**Keywords:** Hip geometry, Total hip arthroplasty, Short-stem design, Straight-stem design

## Abstract

**Background:**

The preservation or restoration of hip geometry following total hip arthroplasty (THA) is of importance, considering that alterations in the centrum-collum-diaphysis (CCD)-angle, femoral offset (FO), acetabular offset (AO) and total offset (TO) change hip biomechanics. Therefore, the most suitable implant should be used. The aim of this study was to compare the ability of two short-stem-systems and one straight-stem-system to reconstruct hip geometry.

**Methods:**

Two-hundred-fifty-one patients (mean age: 62.0 ± 10.0 years; 51.8% males) undergoing THA with three different stem types were retrospectively included, after excluding 11 patients with missing radiological follow-up. Pre- and postoperative radiographic images (group I, *ANA.NOVA Alpha Schaft Proxy®, ImplanTec*, 12 options: *n* = 99; group II, Optimys® *Mathys*, 24 options: *n* = 62; group III: Corail®-System*, DePuy-Synthes*, 76 options: *n* = 90) were analyzed. Differences in pre- and postoperative hip geometry (i.e. CCD, FO, AO, TO) were compared between groups with one-way-analysis-of-variance (ANOVA), and post-hoc t-tests.

**Results:**

The CCD-angle increased by a mean of 8.4° ± 7.2° from pre-to postoperative, with no significant difference between groups (*p* = 0.097). Significantly larger increases in FO were observed for groups II (4.1 mm ± 7.8 mm) and III (4.9 ± 7.2 mm), in comparison to group I (1.6 ± 6.9 mm; *p* = 0.006). AO decreased by a mean of 2.2 ± 4.5 mm, with the largest decrease observed in group III (-3.3 ± 5.3 mm), and the smallest for group I (-1.4 ± 3.6 mm; *p* = 0.011). There was no significant difference in change of TO between groups (*p* = 0.177).

**Conclusions:**

Reconstruction of hip geometry using a single-version novel short-stem-system is achievable with comparable results to stem-systems offering multiple options.

## Introduction

Total hip arthroplasty (THA) has become one of the most successful orthopaedic surgeries of the last century [1]. Considering that the muscle strength following THA is negatively affected by disproportional changes in the lever arm of the *M. gluteus medius*, hip centre and femoral offset, preservation of preoperative hip geometry is of importance [2, 3]. In the past, the main focus was on restoration of leg length, largely neglecting potential changes in the centrum-collum-diaphysis (CCD) angle, femoral offset, and acetabular offset [4]. However, poor hip geometry reconstruction may result in impingement, consecutive polyethylene-wear and aseptic loosening [5–7]. Therefore, stem designs have been adapted over the years, aiming at optimal bony integrity of the implant, good tribological properties, minimal bone resorption and preservation of hip geometry. In order to allow for optimal reconstruction, manufacturers nowadays offer several stem types with varying sizes, lengths, angulations, offsets and depositions. Decision towards a specific implant is not only based on familiarity of staff with instruments and implants, ease of use and surgeon’s preferences, but also on geographical aspects, implant costs, availability, shelf life as well as storage options and spaces [8].

Short-stem designs have gained popularity in THA, with promising short- to mid-term clinical results [9–11]. Apart from the risk of inaccurate implant positioning, postoperative stem migration and intraoperative fractures associated with short-stem devices, hip geometry reconstruction may also be more difficult in comparison to straight stem systems [12, 13]. Due to the predominantly metaphyseal anchorage, the femoral neck has to be partially preserved, which in turn may have a significant influence on postoperative hip geometry, with larger changes in femoral offset, acetabular offset, and centrum-collum-diaphyseal (CCD) angle in comparison to straight-stem devices [9].

Therefore, the aim of the present study was to compare potential differences in hip geometry reconstruction following THA using two short-stem systems and one straight-stem system, taking into account the varying numbers of shaft versions provided for each implant.

## Methods

**Fig. 1:**
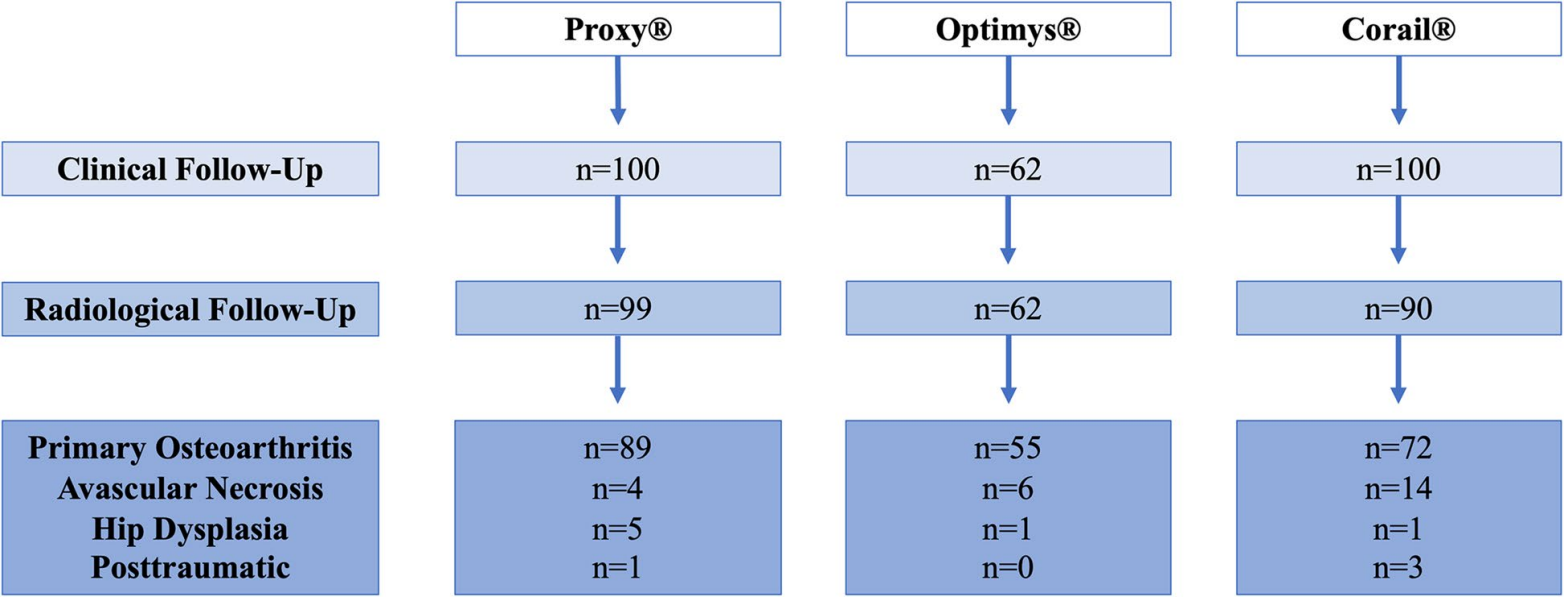
Flow-chart for different stem types

In the present comparative study, 262 patients undergoing THA with three different stem types were retrospectively included. Subsequently, 11 patients with missing radiological follow-up had to be excluded, resulting in 251 patients eligible. Two-hundred-sixteen of these underwent THA for primary osteoarthritis (86.0%), and the remaining patients had secondary osteoarthritis, either due to avascular necrosis (*n* = 24; 9.6%), hip dysplasia (*n* = 7; 2.8%), or preceding trauma (*n* = 4; 1.6%; Fig. 1). Patients had undergone surgery between January 2006 and February 2017 at a single institution, with different shaft types preferably used at specific periods (group III: 2006–2011; group II: 2014–2015; group I: 2016–2017). Surgery had been performed by a single surgeon in groups I and II, whilst several surgeons were responsible for THAs in group III.

All THAs were performed at a single university hospital. As standard procedure, an anterolateral approach to the hip was used. Only in selected cases, a direct lateral approach had been performed. During surgery, prior to wound closure, fluoroscopy was performed in order to confirm correct implant position.

All patients in group I received a cementless short stem with metaphyseal fixation (*ANA.NOVA® Alpha Schaft® Proxy, ImplanTec GmbH, Moedling, Austria*), combined with a cementless press-fit cup (*ANA.NOVA Alpha®, ImplanTec GmbH, Moedling, Austria*). Osteotomy following a partial neck preserving philosophy was used in this group. In group II, all cases of another cementless short stem (*Optimys®, Mathys, Bettlach, Switzerland*) implanted at our institution, combined with the *seleXys® (Mathys, Bettlach, Switzerland*) cementless cup, were included (*n* = 62). Similar to group I, a partially neck preserving osteotomy was carried out. A straight cementless stem (*n* = 90; *Corail® Hip System, DePuy International Ltd., Leeds, England, UK*) was used in group III. The *Corail® Standard KS* had been used in 63 patients (70%) and the *Corail® High Offset KHO* in 27 patients (30%), all with head size 36 mm and without collar, together with the cementless *Pinnacle® 100* cup (*DePuy International Ltd., Leeds, England, UK*). In group III, a trochanter sparing osteotomy was performed. Ceramic-on-ceramic bearings were used in all implant types. In Table 1, available options of the respective stem types are listed.

**Table 1:** Implant details for the three groups

	**Group I** ***N* = 99**	**Group II** ***N* = 62**	**Group III** ***N* = 90**
Stem	***ANA.NOVA Alpha Schaft Proxy®**** ImplanTec GmbH, Moedling, Austria*	***Optimys®*** *Mathys, Bettlach, Switzerland*	***Corail® Hip System*** *DePuy International Ltd., Leeds, England, UK*
Cup	***ANA.NOVA Alpha®*** *ImplanTec GmbH, Moedling, Austria*	***seleXys®*** *Mathys, Bettlach, Switzerland*	***Pinnacle® 100*** *DePuy International Ltd., Leeds, England, UK*
Stem Material	Titanium alloy (TiAI6V4), CaP-coated (BONIT®)	Titanium alloy (TiAI6V4), CaP-coated	Titanium alloy (TiAI6V4), HA-coated
Stem Shape	Triple-tapered	Triple-tapered	Double-tapered
Stem types	Alpha Schaft® proxy	Optimys® Standard Optimys® Lateral	Standard Offset (KS) High Offset (KHO) Collared Standard Offset (KS) Collared High Offset (KHO) Collared Coxa Vara (KLA) 125° Standard 135° Short Neck Collared 125° Standard Collared 135° Short Neck Collared Dysplasia (DDH) Trochanteric Base DDH
Stem options	**12**	**24** 12 Standard 12 Lateral	**76** 13 KS 13 Collared KS 12 KHO 12 Collared KHO 12 Collared KLA 3 125° Standard 3 Collared 125° Standard 3 135° Short Neck 3 Collared 135° Short Neck 1 Collared DDH 1 Trochanteric Base DDH

*Legend: CaP* Calcium-phosphate, *HA* Hydroxyapatite

Follow-up appointments were scheduled at 6 weeks, 6 months, 12 months and annually thereafter with clinical and radiological examination. Demographic data (age at surgery, gender), duration of surgery and pre- as well as postoperative radiographic measurements of hip geometry were ascertained.

**Fig. 2:**
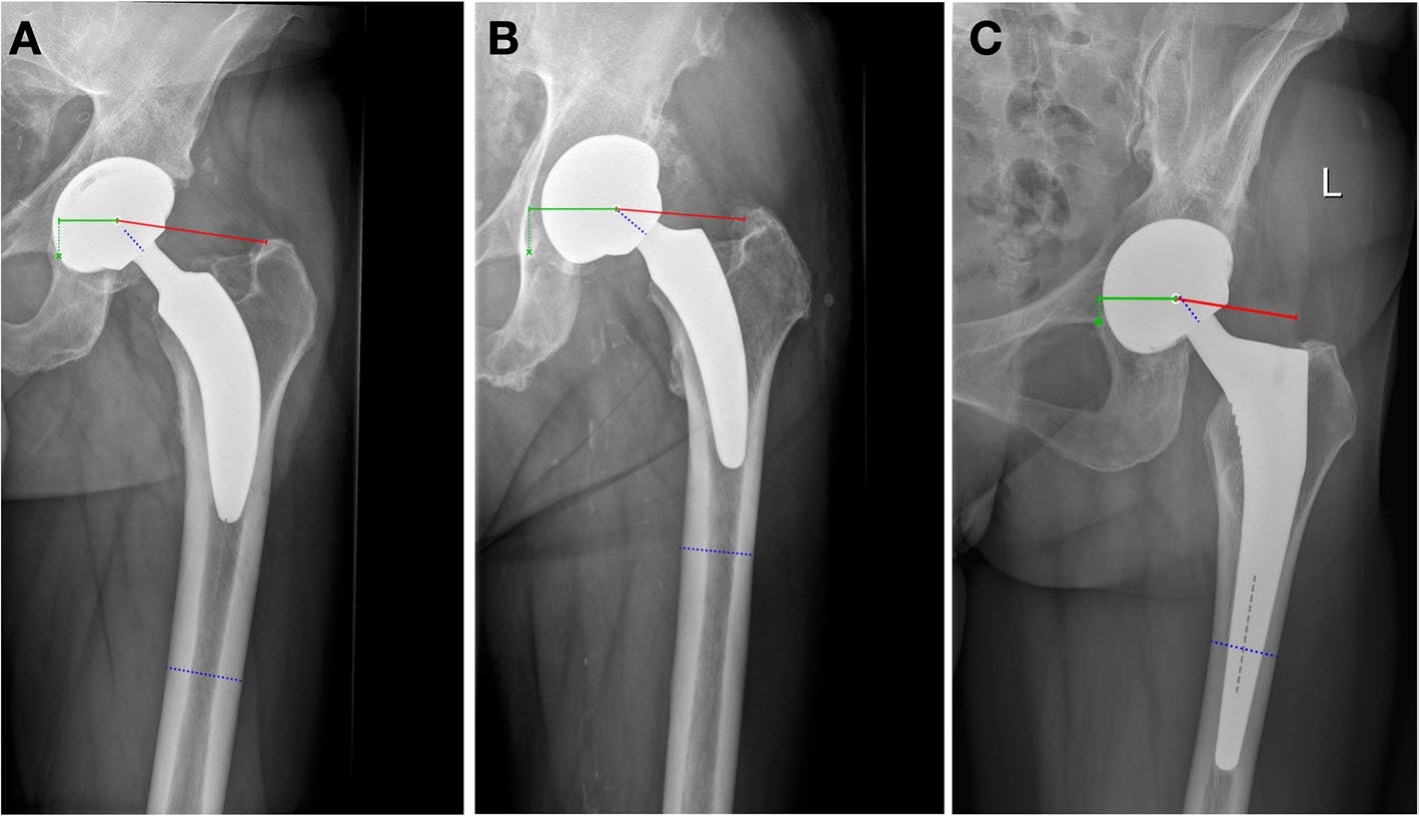
Examples of postoperative hip geometry measurement using the mediCAD® Classic Hip 2D (*Hectec GmbH, Aldorf bei Landshut, Germany*) software for the **A** *Proxy®* used in group I (CCD: 142.3°; FO: 58 mm; AO: 22 mm; TO: 80 mm), **B** Optimys® used in group II (CCD: 146.0°; FO: 51 mm; AO: 34 mm; TO: 85 mm), and the **C** *Corail®* used in group III (CCD: 139.9!; FO: 42 mm; AO: 26 mm; TO: 68 mm). Legend: green line – acetabular offset (AO); red line – femoral offset (FO); dashed blue line + dashed grey line – centrum-collum-diaphysis-(CCD) angle

Preoperative as well as postoperative radiographic images (3 months median time from surgery to image) of the pelvis were used to perform measurements of the CCD angle, femoral offset, acetabular offset and total offset. Radiographic measurements were carried out by two experienced observers using mediCAD® Classic Hip 2D (*Hectec GmbH, Aldorf bei Landshut, Germany*; Fig. 2), who consulted each other in difficult cases. All radiographic images had been performed following a standard protocol, with patients standing straight, feet pointing inwards by 10°, patellae oriented frontwards, and a radio-opaque ball of 25 mm in size centred at the level of the hip joint in order to allow for image calibration in mediCAD®. Femoral offset was measured with a line at 90° to the femoral shaft-axis to the centre of rotation of the femoral head, and the acetabular offset with a line in parallel with the ground to the tear drop figure. The sum of femoral and acetabular offset resulted in the total offset. The CCD angle was measured using a line following the femoral neck as well as a line following the axis of the femoral shaft.

The difference between pre- and postoperative measurements was calculated as postoperative measurement minus preoperative measurement.

### Statistical analysis

Means were reported with standard deviations (SD). Chi-squared tests were used to assess differences in ordinary and categorial variables between groups. Continuous variables were tested for normality using Kruskal–Wallis test. One-way analysis of variance (ANOVA) was performed to assess differences in means between the three stem types. In case of a statistically significant difference with one-way ANOVA, post-hoc t-tests were used to determine in-between variation of means. All analyses were performed with Stata/SE Version 15.1 (*StataCorp, College Station, TX, USA*). A *p*-value of < 0.05 was considered statistically significant.

The current study was approved by the local Institutional Review Board (IRB-Number: 28–152 ex 15/16).

## Results

Ninety-nine patients were included in group I, 62 in group II and 90 in group III. Mean age of all patients was 62.0 years (± 10.0), with those patients in group I being on average 4 and 2 years younger than patients in groups II and III, respectively (*p* < 0.001; Table 2). One-hundred-thirty patients were male (51.8%), and 121 female (48.2%), with no significant difference between groups (*p* = 0.478; Table 2). Mean duration of surgery was 54 min (± 25.6), with surgical time being significantly longer for groups II and III in comparison to group I (*p* < 0.001; Table 2). Further patient-specific parameters are listed in Table 2.

**Table 2:** Differences in patient data and preoperative hip joint geometry between stem types

		**Group I (Proxy®)** ***N* = 99** Count (%)	**Group II (Optimys®)** ***N* = 62** Count (%)	**Group III (Corail®)** ***N* = 90** Count (%)	***P*-value**	***P*-value (t-test)**
**Age** (mean + SD)		60.3 ± 7.8	64.3 ± 10.8	62.3 ± 11.4	**0.047**	I vs II: **0.014** I vs III: 0.177 II vs III: 0.220
**Gender**	**Male**	54 (54.6)	34 (54.8)	42 (46.7)	0.478	
	**Female**	45 (45.4)	28 (45.2)	48 (53.3)		
**BMI** (mean + SD)		28.5 ± 4.9	26.8 ± 4.0	27.6 ± 3.8	**0.047**	I vs II: **0.015** I vs III: 0.133 II vs III: 0.285
**Side**	**Left**	46 (46.5)	30 (48.4)	41 (45.6)	0.942	
	**Right**	53 (53.5)	32 (51.6)	49 (54.4)		
**ASA**	**1**	11 (11.5)	7 (11.5)	7 (7.8)	0.066	
	**2**	60 (62.5)	36 (59.0)	41 (45.6)		
	**3**	22 (22.9)	18 (29.5)	37 (41.1)		
	**4**	3 (3.1)	0 (0.0)	5 (5.6)		
**Primary Osteoarthritis**	**No**	10 (10.1)	7 (11.3)	18 (20.0)	0.115	
	**Yes**	89 (89.9)	55 (88.7)	72 (80.0)		
**Hip Type**	*Coxa vara* (CCD < 125°)	32 (32.3)	23 (37.1)	27 (30.0)	0.131	
	*Coxa norma* (CCD 125°-135°)	43 (43.4)	34 (54.8)	45 (50.0)		
	*Coxa valga* (CCD > 135°)	24 (24.2)	5 (8.1)	18 (20.0)		
**Duration of surgery** (minutes; mean + SD)		40.2 ± 12.5	45.5 ± 15.7	75.4 ± 27.9	** < 0.001**	I vs II: 0.106 I vs III: < **0.001** II vs III: < **0.001**
**Preoperative CCD Angle** (mean + SD; in degrees)		129.3 ± 7.3	127.2 ± 6.1	128.1 ± 7.2	0.167	
**Preoperative Femoral Offset** (mean + SD; in mm)		39.3 ± 7.1	39.0 ± 5.8	36.6 ± 7.4	**0.021**	I vs II: 0.773 I vs III: **0.009** II vs III: **0.042**
**Preoperative Acetabular Offset** (mean + SD; in mm)		36.1 ± 5.0	37.2 ± 3.8	35.2 ± 4.7	**0.040**	I vs II: 0.130 I vs III: 0.228 II vs III: **0.011**
**Preoperative Total Offset** (mean + SD; in mm)		75.3 ± 9.3	76.2 ± 6.4	71.9 ± 8.4	**0.002**	I vs II: 0.545 I vs III: **0.004** II vs III: **0.002**

*Legend: bold* significant result

### Preoperative hip joint geometry

**Fig. 3:**
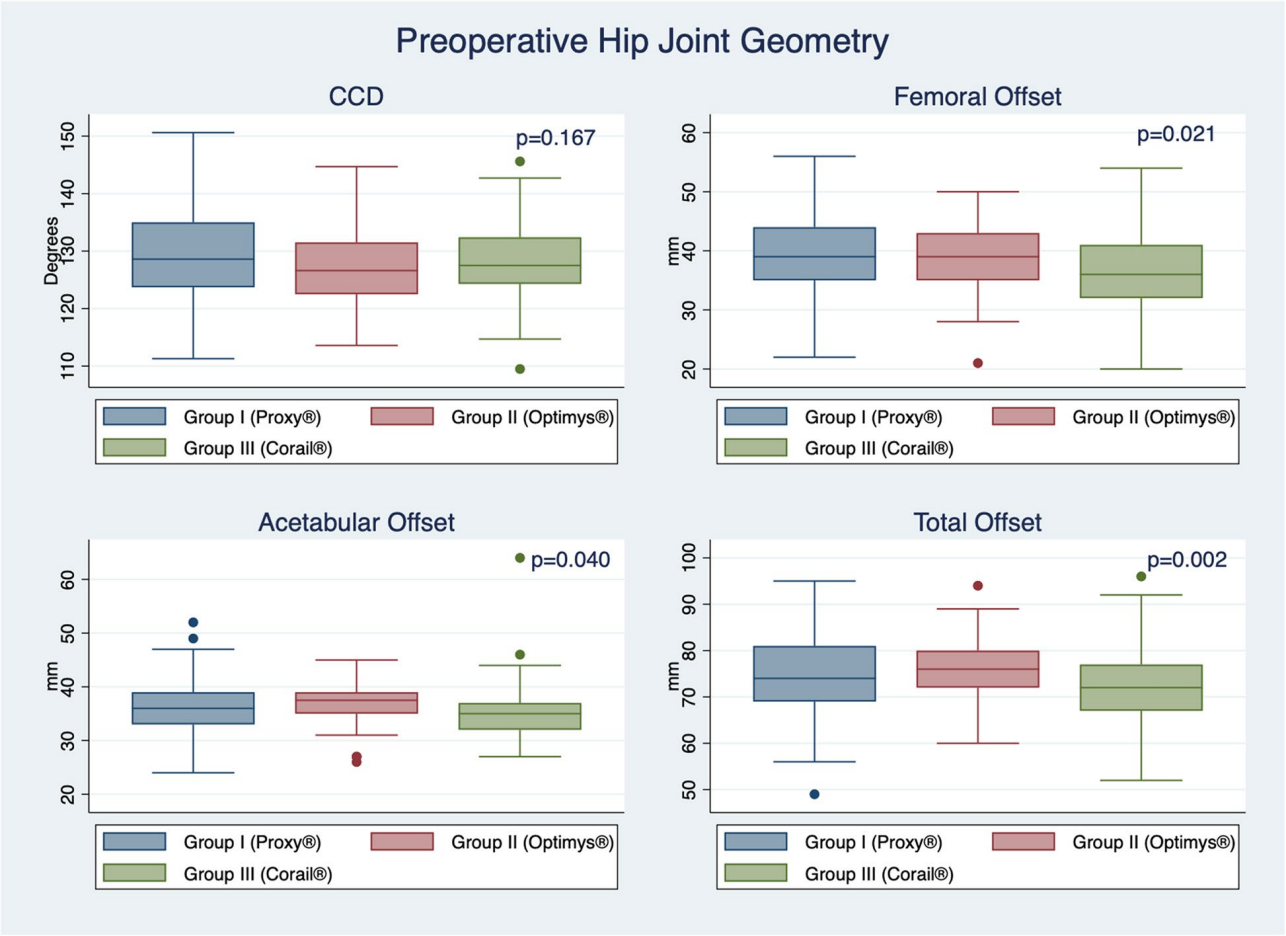
Difference in preoperative hip geometry as assessed on radiographic images by stem types and parameters assessed (*Legend: CCD, centrum-collum-diaphysis angle*)

There was a significant difference in preoperative acetabular offset (36.0 mm ± 4.7 mm; *p* = 0.040), femoral offset (38.3 ± 7.0 mm; *p* = 0.021), and total offset (74.3 ± 8.5 mm; *p* = 0.040) between the three groups, whilst the CCD angle (128.4° ± 7.0°; *p* = 0.167) was comparable (Table 2; Fig. 3).

### Postoperative hip joint geometry

**Fig. 4:**
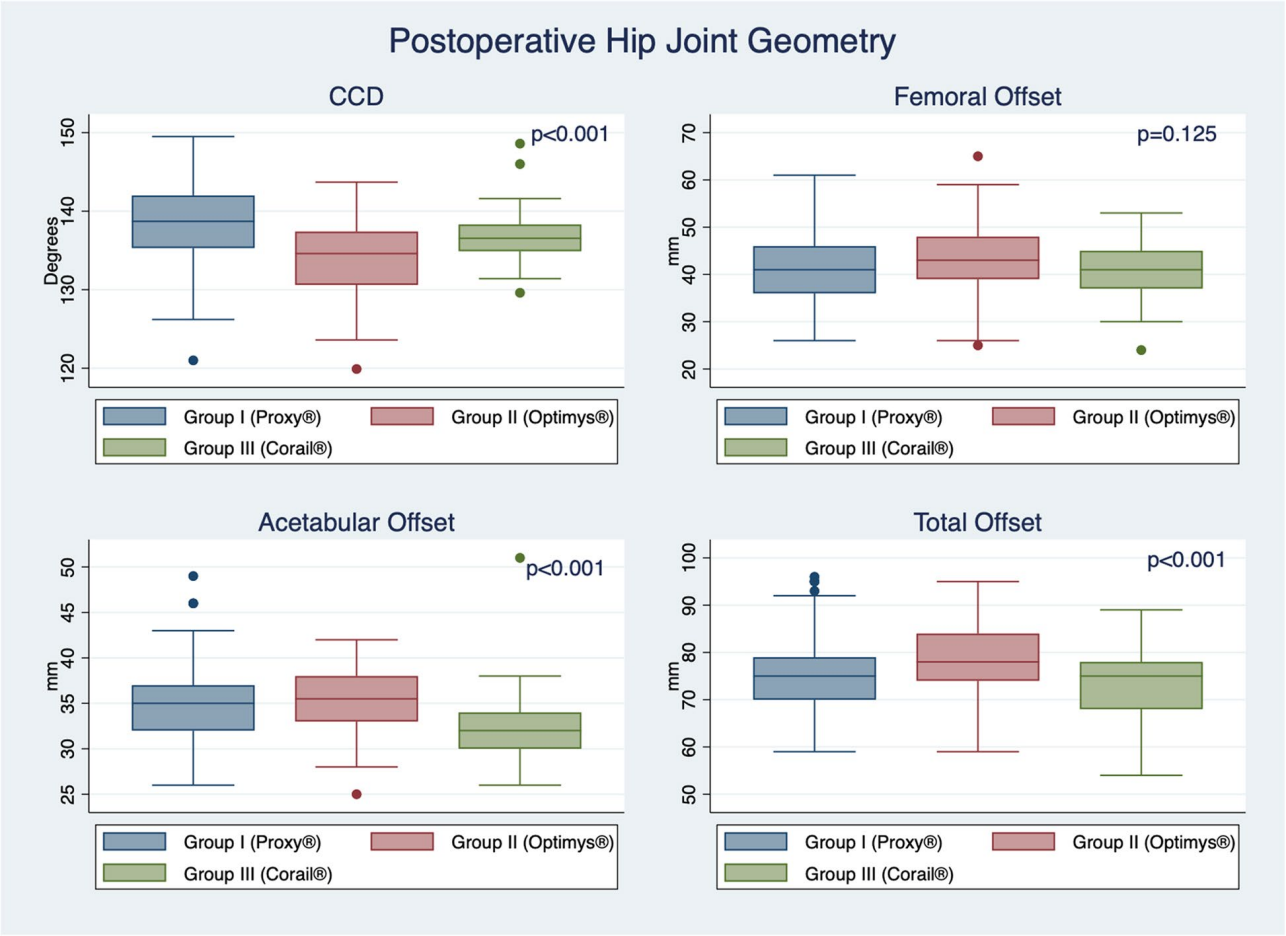
Difference in postoperative hip geometry as assessed on radiographic images by stem types and parameters assessed (*Legend: CCD, centrum-collum-diaphysis angle*)

Postoperative CCD angle did significantly differ between the three groups (*p* < 0.001; Table 3; Fig. 4). Overall, CCD increased by a mean of 8.4° ± 7.2° from pre-to-postoperative, with no significant difference between groups (0.097; Table 3). The femoral offset increased from pre to postoperative by a mean of 3.4 ± 7.4 mm, with the largest increase observed for the straight stem in group III (4.9 ± 7.2 mm), whilst the lowest increase was present for the short stem in group I (1.6 ± 6.9 mm; *p* = 0.006; Table 3).

**Table 3:** Difference in postoperative radiographic measurements between stem types

	**Group I (Proxy®)** ***N* = 99**	**Group II (Optimys®)** ***N* = 62**	**Group III (Corail®)** ***N* = 90**	***P*-value** (one-way ANOVA)	***P*-value (**t-test**)**
**Centrum-Collum-Diaphysis-Angle**					
** Postoperative CCD Angle** (mean + SD; in degrees)	138.6 ± 5.1	134.0 ± 5.0	136.7 ± 2.9	** < 0.001**	I vs II: < **0.001** I vs III: **0.003** II vs III: < **0.001**
** Difference in CCD Angle** (mean + SD; in degrees)	9.3 ± 7.2	6.8 ± 6.9	8.6 ± 7.3	0.097	
**Femoral Offset**					
** Postoperative Femoral Offset** (mean + SD; in mm)	40.9 ± 6.9	43.1 ± 7.4	41.5 ± 5.7	0.125	
** Difference in Femoral Offset** (mean + SD; in mm)	1.6 ± 6.9	4.1 ± 7.8	4.9 ± 7.2	**0.006**	I vs II: **0.033** I vs III: **0.002** II vs III: 0.534
**Acetabular Offset**					
** Postoperative Acetabular Offset** (mean + SD; in mm)	34.7 ± 4.5	35.2 ± 3.6	31.9 ± 3.6	** < 0.001**	I vs II: 0.417 I vs III: < **0.001** II vs III: < **0.001**
** Difference in Acetabular Offset** (mean + SD; in mm)	-1.4 ± 3.6	-2.0 ± 4.4	-3.3 ± 5.3	**0.011**	I vs II: 0.394 I vs III: **0.003** II vs III: 0.071
**Total Offset**					
** Postoperative Total Offset** (mean + SD: in mm)	75.6 ± 7.7	78.3 ± 8.0	73.4 ± 6.4	** < 0.001**	I vs II: **0.024** I vs III: **0.042** II vs III: < **0.001**
** Difference in Total Offset** (mean + SD; in mm)	0.2 ± 6.3	2.1 ± 6.6	1.6 ± 7.2	0.177	

*Legend: bold* significant result

**Fig. 5:**
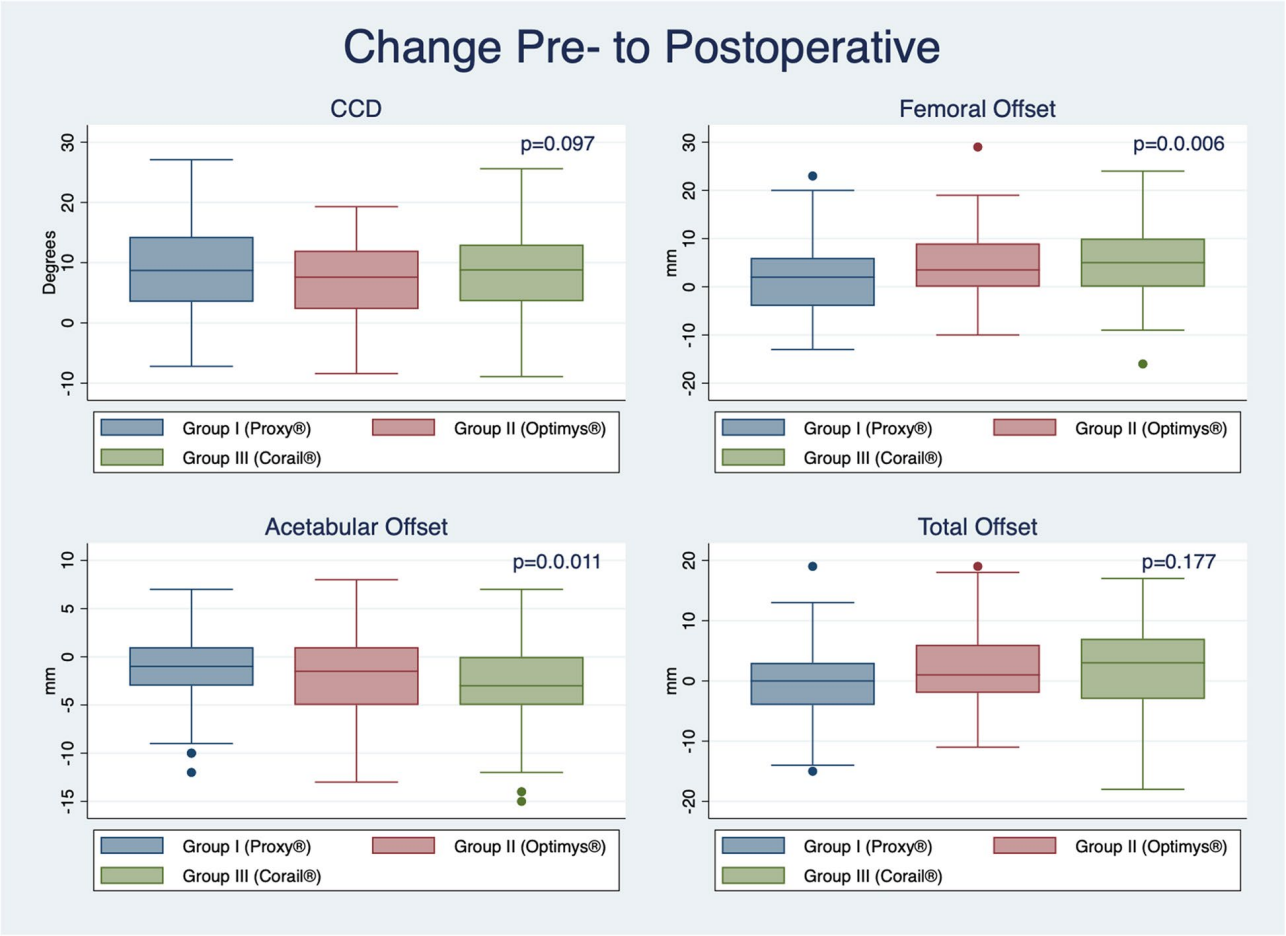
Box plot showing change of pre-to-postoperative hip geometry (CCD-angle, femoral offset, acetabular offset, total offset) by stem group (*Legend: CCD, centrum-collum-diaphysis angle*)

Postoperative acetabular offset was larger in groups I and II in comparison to group III (*p* < 0.001; Fig. 4). Overall, the acetabular offset decreased from pre-to-postoperative by a mean of -2.2 ± 4.5 mm, with a larger decrease for group III in comparison to groups I and II (Table 3). As the femoral offset increased in all groups by a mean of 3.4 ± 7.4 mm, the difference in total offset from pre-to-postoperative was not significantly different between the groups (*p* = 0.177; Table 3). Related to this, Fig. 5 shows the change between pre- and postoperative CCD angle, femoral offset, acetabular offset, and total offset by stem group.

## Discussion

According to the present retrospective cohort study comparing three different stem types, the most accurate reconstruction of hip geometry following THA is possible with an innovative short-stem design in comparison to another short-stem and one straight-stem design. In detail, femoral offset was significantly less changed by the short-stem *Proxy®*, offering 12 options, as compared to the *Optimys®* short stem and the *Corail®* stem offering 24 and 76 stem options, respectively. Additionally, the smallest reduction in acetabular offset from pre- to postoperative was found for group I, being significantly smaller than for the Corail® stem in group III. CCD angle and total offset increased in all three groups, with no statistically significant difference.

One limitation of the present study is its retrospective design, rendering true randomisation of cohorts impossible. Considering that there was no significant difference in baseline parameters as gender and hip type between the three groups, relatively homogeneous cohorts can be assumed. Moreover, surgery had been performed by multiple surgeons in group III, whilst performed by a single-surgeon in the other two groups (I and II). This may have had an effect on longitudinal quality of implant position. Additionally, THAs performed over a relatively long time period were included. However, considering that only the preoperative and immediate postoperative hip geometry had been analysed, this issue may not have had an impact on the observations made. Also, images had been analysed by two reviewers rather than a single one, which may have affected measurements by interobserver variation. As the two reviewers are experienced in measuring hip x-rays and consulted each other in difficult cases, this variation should have been reduced, though. Related to this, the use of x-rays rather than CT scans may have altered some measurements on the 2D image, as CCD angle. Yet, as images were obtained in a standardised manner, variations due to image projection should have been minimised.

One may argue that devices with few stem options had been preferably used in patients with “physiological” hip joint geometry, i.e. those with *coxa norma*, no hip dysplasia, AVN or preceding trauma, thus contributing to favourable results as observed for group I. There was, however, no difference between the three groups regarding hip types (*coxa vara, coxa norma, coxa valga*) and presence of primary vs. secondary hip osteoarthritis.

Our results are comparable to those described by *Innmann *et al., analysing three stems types, i.e. the *CLS® Spotorno®* (*Zimmer Inc., Warsaw, IN*), available in three different neck shaft angles and 13 sizes, the *Profemur® E/EHS implant system* (*European Hip System, Wright Medical Technology Inc., Arlington, TN*), providing 10 sizes in standard or plus-version together with 18 neck options, and the *Fitmore®* (*Zimmer Inc., Warsaw, IN*) short stem, with 14 sizes and 4 angles (allowing for 56 combinations) [14]. According to their study, all three stem designs allowed for good hip anatomy reconstruction. Furthermore, they discovered that neck modularity did not provide significant reconstruction advantages [14]. These observations are in line with our results, considering that despite 76 different options being potentially available in group III, the other two stem types with 12 and 24 options, respectively, did not rank behind in terms of hip joint geometry reconstruction.

The mean increase of femoral offset by 4.1 ± 7.8 mm as observed for the short stem in group II (*Optimys®)* is comparable to the average 5.8 mm increase reported by *Kutzner *et al*.* in a prospective study including 114 patients receiving THA with the same short stem system [4]. For the novel short-stem device (*Proxy®)* investigated in group I, however, a significantly smaller increase in femoral offset was observed in comparison to the *Optimys®* and *Corail®* system. Our results are also contradictory to the observations made by *Schmidutz *et al., comparing the change in hip geometry following THA with a modular short-stem system (*Metha, BBraun Aesculap, Tuttlingen, Germany*) and a straight-stem system (*CR-stem, Implantcast, Buxtehude, Germany*) [9]. In that study, the horizontal femoral offset increased by a mean of 6.2 mm in the short-stem-group, as compared with a mean of 2.0 mm in the straight-stem-group. In our cohort, on the contrary, the smallest increase in femoral offset was observed for the *Proxy®*, being 1.6 ± 6.9 mm in average, as compared to 4.1 ± 7.8 mm and 4.9 ± 7.2 mm for the *Optimys®* and *Corail®* stem, respectively.

Overall, acetabular offset decreased in all three groups, being in line with previous studies [14–16]. Pre- to postoperative differences in acetabular offset were significantly larger for implants in group III (*Pinnacle® 100* cup) in comparison to group I (*ANA.NOVA Alpha®* cup), whilst there was no significant difference for the cup used in group II (*seleXys®*). Notably, changes in acetabular offset rather depend on configuration of the acetabulum, BMI, surgeon’s experience and technique than the cup’s design itself [17, 18].

The CCD angle increased to a comparable amount in all three groups, with a slightly larger change for the *Proxy®* in comparison to the *Corail®* and *Optimys®* stems. This observation may be explained by the fact that preservation of femoral offset requires a greater varus positioning of the prosthesis [19]. Thus far, however, many short-stem systems have failed to preserve the femoral offset due to valgisation of the prosthesis [19–21]. In this respect, the herein investigated *Proxy®* short stem seems to allow for reliable reconstruction of hip geometry, with moderate increase in CCD and concurrent retainment of the femoral offset. Nevertheless, it has to be considered that height and orientation of the osteotomy have a more significant influence on the feasibility of an anatomical hip joint reconstruction using short-stem systems as compared to straight-stem systems.

## Conclusions

The results of our study indicate that the *ANA.NOVA Alpha Schaft Proxy®* short stem, despite offering 12 stem options only, allows for reliable and more accurate reconstruction of hip geometry in comparison to the *Optimys®* short-stem with 24 as well as the *Corail®* straight-stem with 76 options. Although studies with larger patient samples are required to validate our observations, hip geometry reconstruction seems feasible with a novel single-type short stem.

## Data Availability

datasets generated and/or analysed during the current study are not publicly available due to data privacy issues but are available from the corresponding author on reasonable request.

## Abbreviations

ANOVA: Analysis of variance
AO: Acetabular offset
CCD: Centrum-colllum-diaphysis
FO: Femoral offset
SD: Standard deviation
THA: Total hip arthroplasty
TO: Total offset
